# Actomyosin-generated tension on cadherin is similar between dividing and non-dividing epithelial cells in early *Xenopus laevis* embryos

**DOI:** 10.1038/srep45058

**Published:** 2017-03-22

**Authors:** Gaëtan Herbomel, Guillaume Hatte, Julien Roul, Sergi Padilla-Parra, Jean-Pierre Tassan, Marc Tramier

**Affiliations:** 1 CNRS, UMR 6290, Rennes, France; 2 Université de Rennes 1, Institut Génétique et Développement de Rennes, 2 Av du Pr Léon Bernard, 35043, Rennes, France; 3 Microscopy Rennes Imaging Centre, Biosit, Université de Rennes 1, France

## Abstract

Epithelia represent a unique situation where polarized cells must maintain sufficiently strong cell-cell contacts to guarantee the epithelial integrity indispensable for barrier functions. Nevertheless, epithelia must also keep sufficient plasticity which is crucial during development and morphogenesis. Adherens junctions and mechanical forces produced by the actomyosin cytoskeleton are major players for epithelial integrity maintenance and plasticity regulations. To understand how the epithelium is able to meet such a challenge, it is indispensable to determine how cellular junctions and mechanical forces acting at adherens junctions are regulated. Here, we investigate the tensile forces acting on adherens junctions via cadherin during cell division in the Xenopus embryos epithelium. Using the recently developed E-cadherin FRET tension sensor and a fastFLIM prototype microscope, we were able to measure mechanical forces applied on cadherin at cell-cell junctions. We have shown that the Xenopus epithelium is under tension, approximately 3 pN which remains stable, indicating that tensile forces acting on cadherin at the adherens junction are at equilibrium. Unexpectedly, mechanical tension across cadherin was similar between dividing and non-dividing epithelial cells.

Morphogenesis is driven by cellular events such as division, intercalation or extrusion. Actomyosin contractility is a central event in these crucial processes of tissue remodeling. During these processes in epithelia, tissue integrity is constantly preserved by the maintenance of cell-cell contact. To this end, cortical tension and cell-cell adhesion are intimately linked^1^. Localized at the adherens junction, cadherin is a direct molecular connector between neighboring cells^2^. Cadherin is a transmembrane protein whose extracellular domain creates homophilic interactions with cadherin of neighboring cells and which indirectly links, via association of α and β catenins to its intracytoplasmic tail, the actin cytoskeleton^3^.

Traction force microscopy assay using cadherin-coated substrata^4^,^5^,^6^, dual pipette or substrate stretching on cell doublet^7^, or optical tweezers on beads functionalized with cadherin^8^,^9^ have been used to investigate the mechanical roles of cadherin in cells. These studies demonstrate a direct correlation between the extracellular cadherin binding force, the adherens junction size and the generation of force by the cells^4^,^5^,^6^,^7^,^8^,^9^. However, the role cadherin plays in correlating extracellular to intracellular mechanical events remains unclear. Additionally, traction force microscopy experiments demonstrate that α-catenin mediated binding of cadherin to actomyosin is not the sole regulator of cell contractility on cadherin-coated substrata^10^. Another study presents different mechanical roles for P-cadherin and E-cadherin; whereas P-cadherin predicts levels of intercellular force, E-cadherin predicts the rate at which intercellular force builds up^11^. Taken together, the exact role of the cadherin junction appears more complex than just a tension transmitter.

For all the experimental approaches presented above, cell mechanics was tackled at the cellular level and the mechanical role of cadherin at its molecular level was not sensed. The recent approach using FRET tension sensor modules^12^ brings a new vision in cellular mechanics. Using the tension sensor module developed by Schwartz and co-authors for vinculin^13^, tension on cadherin was investigated in the context of substrate stretching on doublet cell adhesion^14^, of border cells migration in drosophila oocytes^15^ and of shear stress on endothelial cells^16^. Using this tool, different tissue-specific isoforms of cadherins were shown to exhibit differential mechanical behaviors. Cortical E-cadherin exhibits increased tension during doublet cell stretching at focal adhesion sites but also out of cell-cell adhesion sites^14^. Inversely, after application of a shear force on endothelial cells, VE-cadherin exhibits a decrease in tension correlated with increased tension across PECAM-1^16^. These initial results do not support a simple molecular mechanical response of cadherin through external or internal application of tension.

During cell division in epithelia, membrane deformation occurs during cytokinesis at the adherens junction. It has been intensively investigated in drosophila^17^,^18^,^19^,^20^, but the mechanisms by which this process is driven, in term of molecular tension and membrane deformation, are not well understood^21^,^22^. Is cadherin stretched by actomyosin at adherens junction to create membrane tension and deformation? Is cadherin implicated in the stability of cell-cell adhesion to preserve the tissue integrity? Can cadherin simultaneously play these two roles? To elucidate these questions, we have investigated tension applied on cadherin during cell division in Xenopus embryo epithelium. At blastula stage, *in situ* observation of cell division is possible since polarized epithelial cells divide with a high frequency at the surface of the organism^23^. We have employed the approach of E-cadherin FRET tension sensor^14^ in developing *Xenopus leavis* embryos. Using the fastFLIM prototype developed in the team^24^ for direct measurement of FRET efficiency, we were able to measure mechanical forces applied on cadherin at cell-cell junctions. Our results show that, at the end of blastula stage, actomyosin-generated tension on cadherin is approximately 3 pN and remains stable during epithelial cell division.

## Results

### Cadherin Tension Sensor exhibits decrease in FRET at cell-cell junction in living *Xenopus* embryo, compared to tail less control

**Figure 1:**
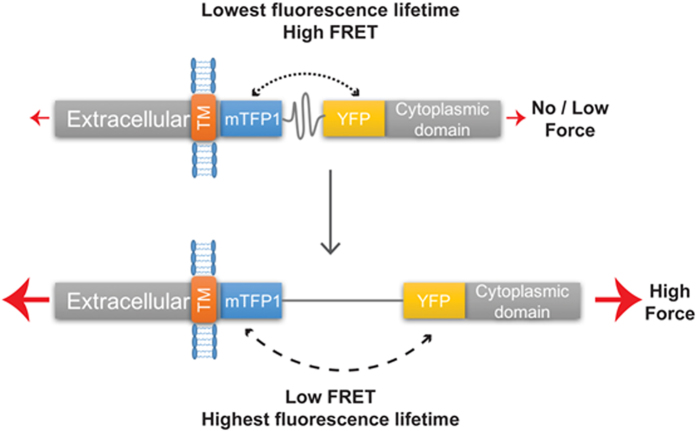
Relation between FRET, fluorescence lifetime, and force applied to the EcadTSMod biosensor. Schematic representation of the EcadTSMod biosensor. In absence of tension (top) the TSMod is not elongated, the distance between the two fluorescent proteins is the shortest, meaning that the FRET is maximum. As the relation between the FRET and donor fluorescence lifetime is inverse, in absence of tension, the donor fluorescence lifetime is low. In presence of force (bottom) the TSMod is elongated, the distance between fluorescent proteins increases, the FRET decreases and the donor fluorescence lifetime increases.

**Figure 2:**
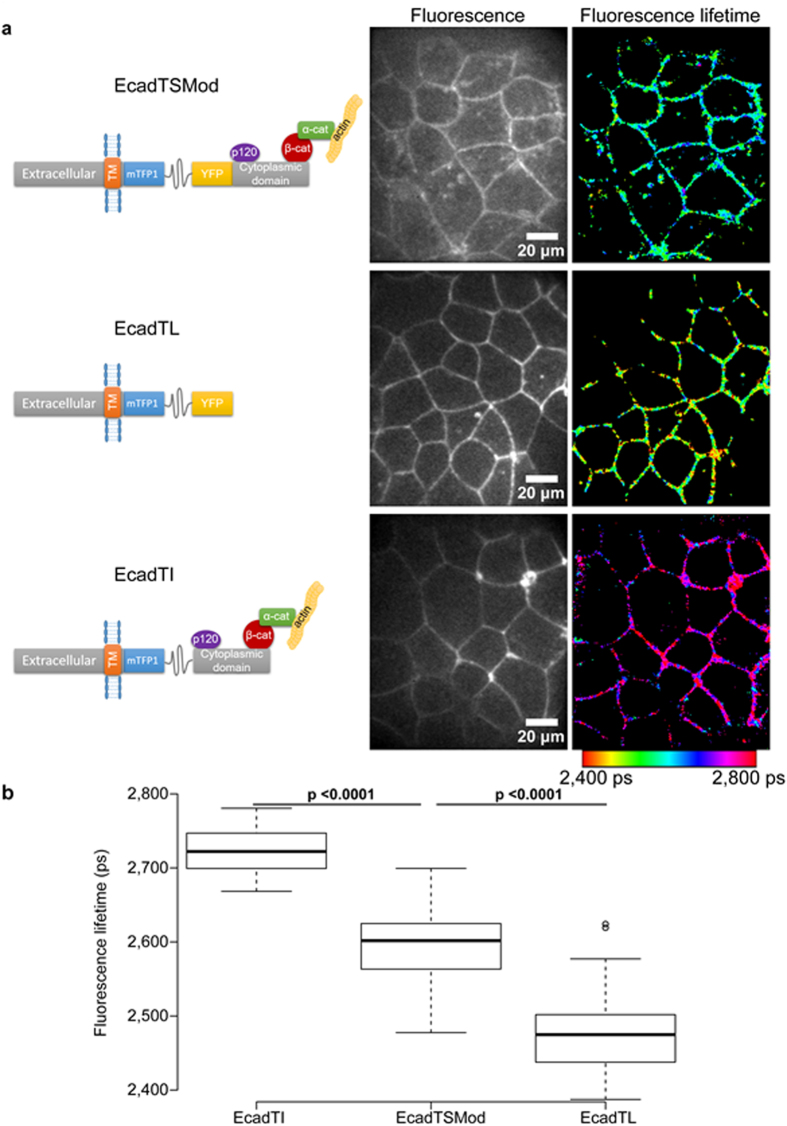
E-cadherin tension sensor displays a FRET decrease in living Xenopus embryo compared to tail less E-cadherin. (**a**) Schematic representations of the different constructions used, showing binding regions of p120 catenin, β-catenin and indirect binding of α-catenin and actin, and their respective localization in epithelial cells of Xenopus laevis blastula (fluorescence), each field of view is composed of about 15 to 20 cells at this stage of development. The fluorescence lifetime images are presented for all constructions (fluorescence lifetime). (**b)** Boxplot representative of the mean fluorescence lifetime per field obtained over all acquisitions for the various constructs. Number of acquisition N_acq_ = 48, 105 and 44, number of independent experiment N_exp_ = 10, 14, 6 and number of embryos N_emb_ = 22, 55, 15, for respectively EcadTI, EcadTSMod and EcadTL. For each comparison, the p value is indicated. In the box plot, bold bars correspond to median value, whiskers to the value 1.5x away from the 1^st^ and 3^rd^ quartiles, and the dots to outliers.

To investigate mechanical forces applied to cadherin at cell-cell junction in epithelial cells of Xenopus embryo, we used the E-cadherin tension biosensor (EcadTSMod, Figs 1 and 2a) previously developed by Borghi *et al*.^14^. The implementation of the tension sensor module (TSMod) between the transmembrane domain and the p120 catenin binding domain did not affect cadherin localization in the Xenopus embryo (Fig. 2a). In this study, we measured FRET by Fluorescence Lifetime Imaging Microscopy (FLIM). The interest in using FLIM for biosensor analysis over ratiometric analysis resides in the fact that fluorescence is measured only for the donor protein, thus eliminating possible spectral bleed-through coming from imaging of donor and acceptor fluorescent proteins. For this purpose, we developed a FastFLIM microscope^24^ which is less invasive compared to other FLIM devices such as TCSPC, and acquires fluorescence lifetime images in a few seconds. The fluorescence lifetime of the donor protein is affected by the FRET phenomenon. The relation between fluorescence lifetime and FRET is inverse, meaning the more decrease in fluorescence lifetime the more FRET occurs, and inversely (Fig. 1). In the example of the TSMod, in absence of force, the FRET is maximum, this corresponds in FLIM to a low fluorescence lifetime compared to the donor alone. In presence of force, the FRET decreases, therefore the fluorescence lifetime increases up to the value of the donor alone. The mean fluorescence lifetime of EcadTSMod measured with the FastFLIM microscope was of 2,594 ± 51 ps (Fig. 2a,b). The fastFLIM system is based on a spinning disk, so that the measure performed was restricted to the the adherens junctions, located in the apical pole of the cells. As expected, the fluorescence lifetime of cadherin donor alone (EcadTI) which lacks the YFP fluorescent protein was significantly higher, at 2,724 ± 31 ps (Fig. 2a,b). This result indicates that the EcadTSMod is capable of FRET in the embryo. Importantly, the fluorescence lifetime of the cadherin tail-less construct (EcadTL) in which the E-cadherin cytoplasmic tail responsible for actin attachment is missing was of 2,476 ± 52 ps (Fig. 2a,b). This fluorescence lifetime was significantly smaller than EcadTSMod thus indicating a modulation of FRET efficiency induced by the linkage of EcadTSMod with actomyosin cytoskeleton. It also suggests that in the Xenopus embryo, mechanical forces produced by the actomyosin cytoskeleton could be involved in force production applied on EcadTSMod at the adherens junction, as previously shown in MDCK cells^14^.

The EcadTSMod biosensor was developed from canine E-cadherin. Modeled on EcadTSMod, we constructed a CcadTSMod biosensensor by inserting the TSMod in the Xenopus C-cadherin which is maternally expressed and is the major cadherin in the early embryo^25^. Localization of CcadTSMod was the same as EcadTSMod (compare Fig. S1a with Fig. 2a). The fluorescence lifetime of CcadTSMod was 2,616 ± 53 ps (Fig. S1b). This result shows that the heterologous EcadTSMod and the homologous CcadTSMod display similar fluorescence lifetimes. Therefore, subsequent experiments were performed with the EcadTSMod and validated with the CcadTSMod as indicated.

### Cadherin tension sensor is able to measure tension variation *in vivo*

**Figure 3:**
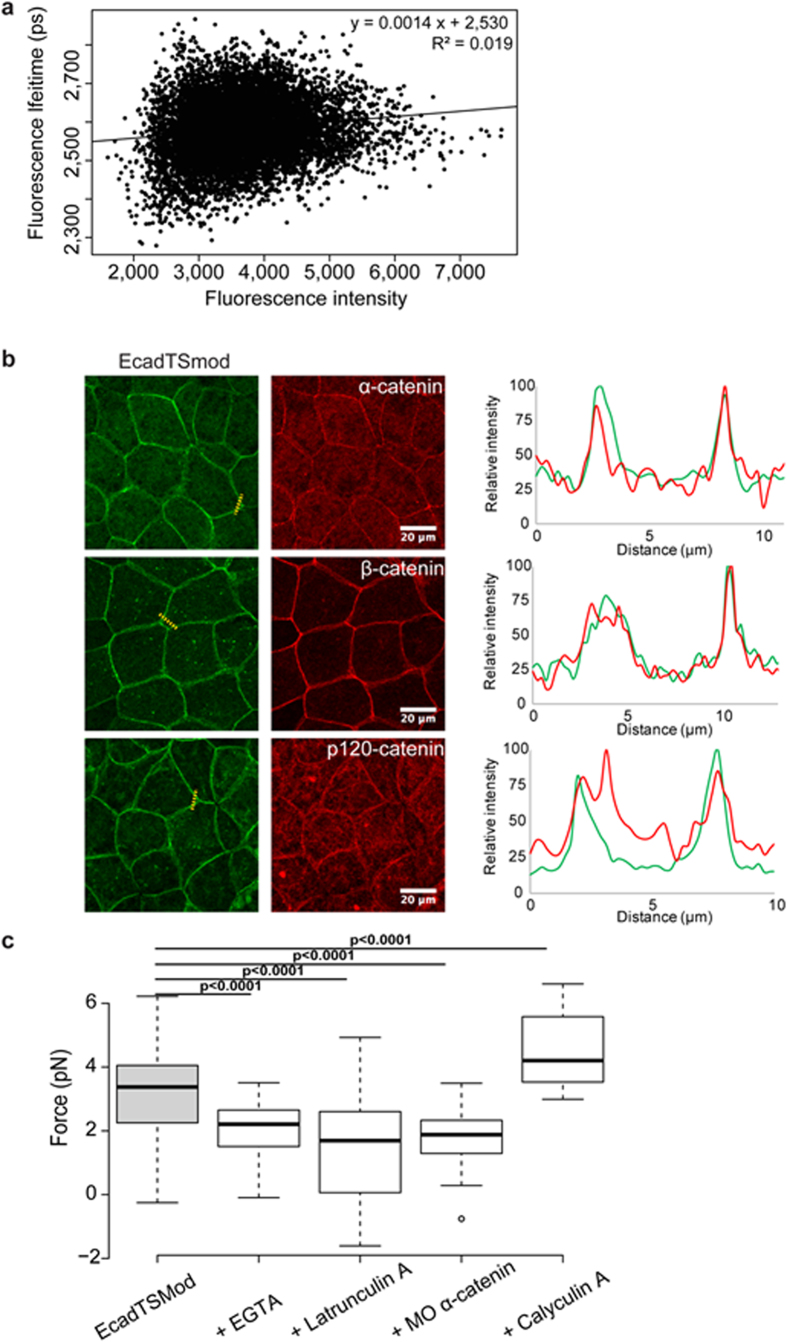
EcadTSMod biosensor is integrated into the adherens junction and responds to several treatments. (**a**) Scatter plot of the fluorescence lifetime vs fluorescence intensity for each pixel of a representative acquisition. Equation of the linear regression is specified in the top right corner. (**b**) Indirect immunofluorescences of EcadTSMod detected with anti-GFP antibody (green) and α, β and p120 catenins (red) in Xenopus laevis blastula overexpressing the EcadTSMod protein. Linescan were made on the indicated dotted line, and the normalized fluorescence intensity is shown for both channel (green for EcadTSMod and red for catenins) in the corresponding graph. Scale bar, 20 μm. (**c**) Boxplot of mean forces applied on EcadTSMod in untreated embryos or embryos treated with EGTA, latrunculin A, morpholino against α-catenin (MO α–catenin) and Calyculin A, with respectively 105, 32, 37, 44 and 29 independent fields (respective number of experiments: 14, 2, 2, 2 and 3; respective number of embryos: 55, 16, 19, 22 and 15). For each comparison, the p value is indicated. In the box plot, bold bars correspond to median value, whiskers to the value 1.5x away from the 1^st^ and 3^rd^ quartiles, and the dot to outlier.

As determined previously by single-molecule stretching, the elastic linker inserted between mTFP1 and YFP in the TSMod, is responsive for forces ranging from 0 to 7 pN^13^. However, the spatial resolution of our FastFLIM system allowed measuring the mean fluorescence lifetime of several molecules localized at the same time in the same volume of acquisition. This raised the two following possibilities: the mean fluorescence lifetime that we measured could actually result from multiple EcadTSMod molecules submitted to a range of forces comprised between 0 and 7 pN or from two distinct EcadTSMod populations. Among these two populations, one population would be under maximal tension (ON) and a second one under no tension at all (OFF). To test these two possibilities, we analyzed the correlation between fluorescence lifetime and fluorescence intensity. Indeed, if the mean FRET reflected a real force then the fluorescence lifetime should not depend on the fluorescence intensity. Conversely, if the FRET reflected a mixture of ON and OFF states, then the two quantities should be different following the level of expression of the sensor and the measured lifetime should depend linearly on the fluorescence intensity. One representative curve of the fluorescence intensity versus the fluorescence lifetime of a field is presented in Fig. 3a. The mean equation obtained for 10 independent experiments is y = 0.0037x + 2,548 and the mean R^2^ value is 0.013. So the vast majority of the fluorescence lifetime data are not correlated to the fluorescence intensity. A similar result was obtained with the CcadTSMod (Fig. S2a). Our results support the hypothesis in which the mean fluorescence lifetime results from multiple molecules subjected to a range of forces comprised between 0 and 7 pN. Therefore suggesting that the EcadTSMod biosensor is subjected to tensions in the embryo and it does not depend on the quantity of cadherin integrated in the junction.

To further show that the EcadTSMod biosensor is well integrated in the junction and associates with its regulators, we analyzed the expression of α, β and p120 catenins in cells overexpressing EcadTSMod. Line scans across different cell-cell junctions present simultaneous recruitment of EcadTSmod and the different catenins (Fig. 3b). Catenins are properly localized at the junction when EcadTSmod is expressed. Therefore, our results indicate that the biosensor is a meaningful tool for force measurements *in vivo*.

Next, to convert fluorescence lifetime into force intensity, we adapted the calibration used by Grashoff *et al*.^13^, and adapted it to FLIM measurement using the FRET fluorescent protein couple mTFP1/YFP. The calculation process is detailed in supplementary material and the resulting calibration curve is shown in Fig. S3a. The resulting calibration equation was y = 0.0292x − 72.597, where y indicates the force in pN and x the fluorescence lifetime expressed in picoseconds. This equation allowed calculating the mean force applied across EcadTSMod in Xenopus embryos (Fig. S3b), with a value of 3.16 ± 1.50 pN (Fig. 3c).

To test if EcadTSMod was able to detect variations in force intensity *in vivo*, we conditionally modified the tension in Xenopus embryos and then measured the impact of such tension changes on EcadTSMod fluorescence lifetime. Because, cadherin is a transmembrane protein linked to cadherin of neighboring cells via its extracellular domain and to the actin cytoskeleton via the association of catenins on its intracytoplasmic tail, there are two ways to modify forces *in vivo*: either by acting on the extracellular cadherin-cadherin interaction or on the actin linkage.

As cadherin is a calcium-dependent adhesion protein, embryos expressing EcadTSMod were treated with 3 mM EGTA to disrupt homophilic interactions. After 20 minutes of treatment, a local cell bowing and in some cases a separation of neighboring cells could be observed (not shown). Those embryos were discarded to analyze only embryos in which the cell-cell interface was not disrupted. As expected, a decrease in force intensity applied to EcadTSMod was measured, with a mean value of 2.04 ± 0.87 pN (Fig. 3c). Similarly, CcadTSMod fluorescence lifetime was reduced (Fig. S2b).

Because the force exerted on the EcadTSMod sensor is also dependent on the intact actin cytoskeleton^14^, we sought to decrease force intensity by using latrunculin A to disrupt actin filaments. Embryos expressing EcadTSMod treated with 5 μM latrunculin A, showed a decrease of force intensity with a mean value of 1.50 ± 1.65 pN (Fig. 3c). As exposure of the embryo to Latrunculin A may induce a loss of cell-cell adhesion as for EGTA treatment, we analyzed only the embryos with intact cell-cell interface. We did not observe a change in fluorescence lifetime at any time after treatment (from 5 to 30 min, data not shown). The same results were obtained for CcadTSMod (Fig. S2b). The residual force that we still observed might result from a residual presence of actin filaments, as the treatment with latrunculin A was not totally effective. Indeed, immunofluorescence of actin after latrunculin A treatment showed a decrease of actin cytoskeleton staining, but some actin filaments were still detected (Fig. S4).

In addition to EGTA and latrunculin A treatments, we also knocked down the expression of α-catenin. Because α-catenin indirectly links cadherin to the actomyosin cytoskeleton, a decrease of α-catenin at the junction should induce a decrease of tension due to a loss of linkage of cadherin to the actin cytoskeleton. We used a previously described morpholino against α-catenin, shown to reduce effectively the α-catenin expression in xenopus embryos^26^. In our hands, Embryos co-injected with both EcadTSMod and morpholino against α-catenin displayed a significant decrease of α-catenin expression (Fig. S5). As expected, the decrease of α-catenin expression was accompanied by a significant decrease of force (Fig. 3c) with a mean value of 1.78 ± 0.76 pN. This effect was also obtained for CcadTSMod (Fig. S2b).

These experiments show that by decreasing actin contractility or by disrupting cadherin linkage we can measure the force decline using the EcadTSMod biosensor. To show that we were also able to measure an increase in tension, we treated the embryos with Calyculin A. Calyculin A is a phosphatase inhibitor that inhibits the myosin-light-chain phosphatase, which induces a hyper-activation of the myosin and leads to increase contractility^27^. Treatment of embryos expressing EcadTSMod with 0.5 μM Calyculin A for 90 minutes induced a significant increase in tension (Fig. 3c), with a mean value of 4.52 ± 1.20 pN. The same effect was obtained for CcadTSMod (Fig. S2b). These results show that the EcadTSMod and CcadTSMod biosensors detect decreased and increased tension.

### Tensions across cadherin are similar in non-dividing cells and during cell division

**Figure 4:**
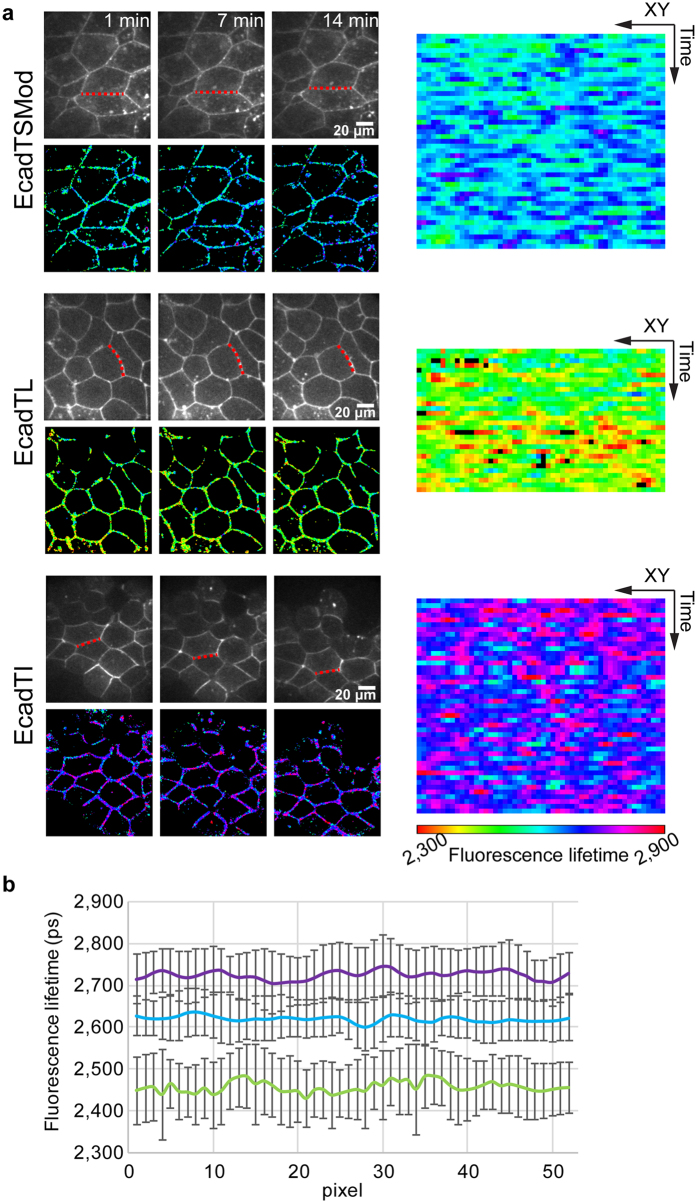
Spatio-temporal analysis of E-cadherin biosensor in the Xenopus embryo. (**a**) Temporal acquisition of EcadTSMod, EcadTL and EcadTI in Xenopus blastula. Fluorescence images are presented in the upper row; the lower row corresponds to fluorescence lifetime images. For each construct, 3 time points are presented 1, 7 and 14 minutes. A kymograph of the whole temporal acquisition is represented in the right part, the corresponding XY coordinates are shown by the red dotted line in fluorescence images. (**b**) Mean fluorescence lifetimes and standard deviations of all XY coordinates over the time series for EcadTI (purple), EcadTSMod (cyan) and EcadTL (green). Values represent the mean ± SD.

A main advantage of our FastFLIM prototype was that it allows carrying out FLIM images in a few tens of seconds. We therefore used FastFLIM to make temporal measurement of fluorescence lifetime in living embryos to detect forces over time. To this end, the fluorescence lifetimes of EcadTSMod, EcadTL and EcadTI were measured over several minutes, typically 15 minutes and for each construct. The fluorescence lifetimes fluctuations over time of typical junctions were shown as kymographs (Fig. 4a, dotted red lines). For each kymograph, there was a high spatial and temporal variability of the fluorescence lifetime. However, the mean fluorescence lifetime was stable along the analyzed membrane over time (Fig. 4b) and representative of the mean value shown previously (Fig. 2). The same analysis was conducted with CcadTSMod (Fig. S6a) and showed that the fluorescence lifetime was also stable over time at the cell-cell junction (Fig. S6b). The standard deviation reflects the error of the measurements and displays the spatial variability of the fluorescence lifetime. This value was similar for the three constructs meaning that it was not condition-dependent. Importantly, the intra-embryo variability was similar for the three constructs, potentially reflecting a variability coming from the fastFLIM custom-built setup. Therefore, we conclude that any changes included into that variability could not be attributed to mechanical tension variations. The standard deviation shown here reflects the intra-embryo variability, while the one shown in Fig. 2 reflected the inter-embryo variability. Both standard deviations were similar indicating that the inter-embryo variability might mainly take into account this intra-embryo variability. In conclusion, our results show that the measurement of forces applied on EcadTSMod or CcadTSMod are stable over time in blastula, indicating that the cadherin tension on the cell-cell junction is at the equilibrium in the embryo.

**Figure 5:**
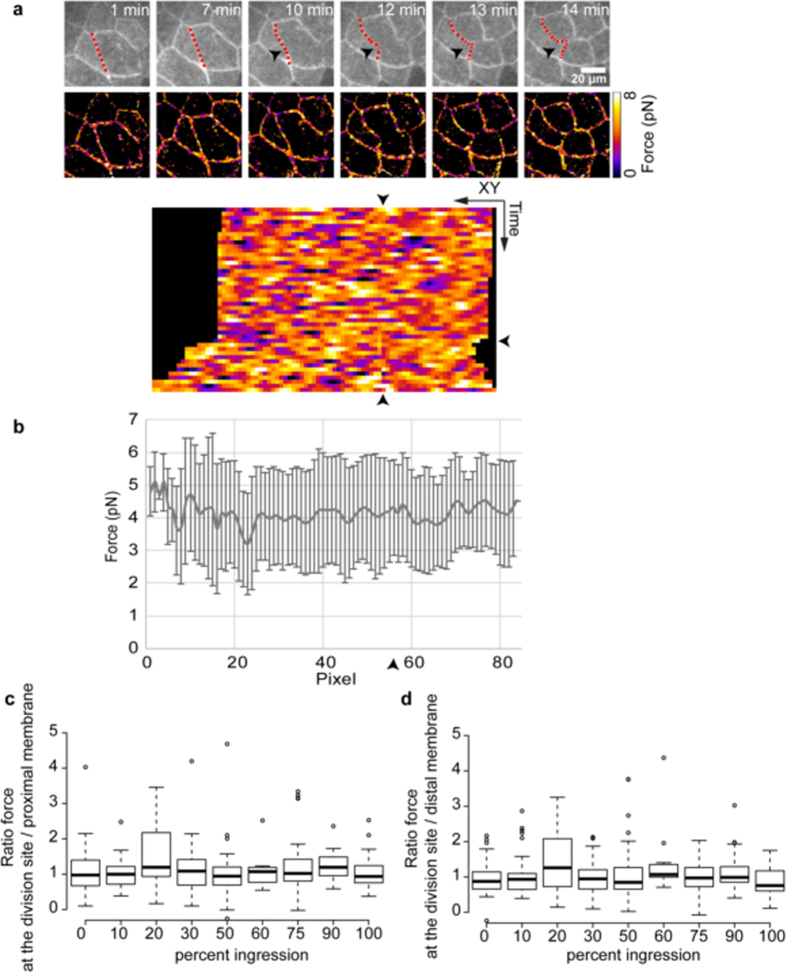
Forces applied to EcadTSMod are stable during epithelial cell division. (**a**) Spatio-temporal acquisition of EcadTSMod fluorescence images and the corresponding force images at different time points. On fluorescence images, the black arrowheads point to the division site and the red dotted lines indicate the cell-cell contact presented as a kymograph below. The temporal resolution is 1 image every 20 seconds. The vertical arrowheads on the kymograph indicate the site of division while the horizontal one marks the starting time of contraction. The length of the kymograph varies over time due to the stretching of the membrane during the division. (**b**) Mean forces and standard deviations of all XY coordinates over the time series for EcadTSMod in dividing Xenopus embryo epithelial cells, the arrowhead indicates the site of division. Values represent the mean ± SD. (**c**,**d**) Ratio of the force measured at the dividing site and on the proximal membrane (**c**) or on a distal membrane on a non-dividing cell (**d**), during the progress of cell division. The force is measured at the same time point at the dividing site and in a region in the proximal or distal membrane. The corresponding percentage of progress of division is calculated by measuring the distance of the two dividing point. The graph shows the results obtained for 44 division sites (which represents 7 independent experiments, 18 embryos and 27 cells). In the box plot, bold bars correspond to median value, whiskers to the value 1.5x away from the 1^st^ and 3^rd^ quartiles, and the dots to outliers.

As the force remains stable over time, we tested if the intensity of forces applied to cadherin changes during cell division. Indeed, as the cytokinetic actomyosin ring positioned perpendicular to the epithelium plane constricts, it produces cell-cell junction deformation and allows plasma membrane ingression. This ultimately leads to the formation of new membranes and junctions between the two daughter cells and between daughter cells with their neighbors. The mean force value of entire fields of acquisition in which cells are dividing (3.50 pN ± 1.72, n = 30) was similar to the one in fields without dividing cells (2.81 pN ± 1.68, n = 42, pvalue = 0.1089). In cells undergoing cytokinesis we analyzed, over time, the tension specifically applied on the plasma membrane which is stretched by actomyosin contraction. A representative result is shown in Fig. 5a. As in the case of non-dividing cells, EcadTSMod force did not change significantly even at the site where the membrane is stretched. The same analysis was performed with EcadTL (Fig. S7a and c) and EcadTI (Fig. S7b and c) controls, in both case the fluorescence lifetime was stable along the membrane and at the division site. For EcadTSMod, the mean force applied during cell division and the standard deviation were similar along the various pixel of the membrane (Fig. 5b) and at the site of division (arrowhead) over time for the representative experiment. The standard deviation here showed the variation over time for a given position on the membrane, therefore we saw no changes in the amplitude of the variation at the dividing site (arrowhead) and elsewhere in the membrane. The same analysis was performed for CcadTSMod (Fig. S8a and b) and showed that the fluorescence lifetime was also stable during cell division when CcadTSMod is expressed.

Using EcadTSMod we analyzed the force during cell division, for the 27 cell division data, in three different regions; at the division site, a proximal region on the membrane undergoing ingression, and a distal region on membrane of a non-dividing cell. The ratio of the force at the dividing site and on proximal membrane is shown in Fig. 5c. During cell division, the ratio was close to 1, with median values between 0.93 and 1.19, and no significant variations were observed. This means that the force at the division site is similar to the one applied on the same membrane at regions that do not undergo ingression. Conversely, the normalized force obtained for embryos treated with the tension-inducer Calyculin A (Calyculin A/non treated embryos, Fig. 3c), shows median values of 1.43 which is significantly higher than what observed with EcadTSmod during cell division. This confirms the absence of strong tension at the division site. The same result was obtained when comparing the force with a region on a distal membrane on a non-dividing cell, with median value included between 0.76 and 1.26 (Fig. 5d), meaning that the fluorescence lifetime observed at the division site is also similar to the one obtained on membrane of cells that do not undergo division. Similar analysis was performed for CcadTSMod, using the fluorescence lifetime values (Fig. S8c and d for 11 cell division data), and also shown for this constructs that the ratio was close to 1 for proximal and distal membrane and did not change over the progress of cell division. These results show that during cell division, the forces across EcadTSMod and CcadTSMod biosensors are similar to non-dividing cells. It also demonstrates that actomyosin-generated tension on cadherin is stable in the developing embryo including during the cytokinesis process.

## Discussion

Using the EcadTSmod tension sensor described previously^14^ and our CcadTSMod, we have shown that cadherin is under tension in the Xenopus blastulae epithelium and that the tensile forces are generated by the actomyosin cytoskeleton. We have also shown that the tension is stable over time, indicating that in the embryo the overall tension applied on cadherin is at the equilibrium. Moreover, we show that during cell division mechanical forces applied on cadherin do not change significantly.

Using anti-C-cadherin morpholinos which successfully depletes the protein in gastrulae^28^ we were unable to knock down C-cadherin expression in blastulae, presumably due to the C-cadherin protein stockpile already present in the egg^25^,^29^. This prevented us from directly testing the functionality of the tension sensors in cadherin-depleted embryos. Both EcadTSMod and CcadTSMod integrate into the junction of living Xenopus embryo and do not disrupt the localization of catenin proteins essential for their function and respond to various stimuli. Therefore, our data suggests they are fully functional. Surprisingly, none of the various treatments used to alter force intensity were able to completely eliminate force as measured by the EcadTL construct. This could simply be explained by the partial effects of latrunculin-A, EGTA or α-catenin morpholino treatments. Nevertheless, those treatments significantly decreased the force applied on EcadTSMod and CcadTSMod, thus showing that these probes are indeed under mechanical tension in the epithelium of Xenopus embryo.

Except for the variability around the mean force value, we could not detect significantly higher variations. We cannot exclude the possibility that small variations could occur but they would not be measurable as they would be included in the measurement error. To detect such tiny variation would require an even more sensitive and faster microscope setup. Thus, our results indicate that the force applied to cadherin in the blastula embryo is stable. The mean tension across cadherin in the Xenopus embryo epithelium measured with the two tension biosensors (ECadTSMod and CCadTSMod) is around 3 pN. Interestingly, this value is only slightly higher than the 1–2 pN measured with ECadTSMod in mammalian cultured cells^14^. This small difference could be due to experimental variation. However, stretching a pair of cells generated a modest but significant 1 pN increase of force at the cell-cell contact^14^. Therefore, the 1–2 pN higher value of force intensity measured in the Xenopus embryo epithelium could reflect a significant difference between the two biological systems. Indeed, it is also possible that the adherens junction of cells of a continuous epithelium such as the one at the surface of the Xenopus blastulae is submitted to more intense forces (superior than 7 pN and thus not detectable with probes optimized for a 0–7 pN range, as it is the case for EcadTSMod and CcadTSMod). To have a clear understanding of how tissue and cell environment influence forces applying to cell-cell junction, future studies will be necessary to compare different type of epithelium or new tensions sensors.

As the tension sensor could be used to measure force *in vivo*, we sought to analyze forces applied at the adherens junction during cytokinesis, the last step of cell division. Cytokinesis involves a contractile acto-myosin ring which deforms the plasma membrane and the associated cytoskeleton cortex thus ultimately leading to separation of the two daughter cells. To test the possibility that forces applied to the adherens junction through cadherin would change during cell division in the embryo epithelium, we measured tension using the two biosensors previously validated. Except variation inherent to the measurements, we did not detect significant changes to the tension along the membrane meaning that cadherin is submitted to tensile forces which are at equilibrium.

Very interestingly, our results indicate that during cytokinesis in the Xenopus embryo epithelium, membrane ingression does not involve major changes in tensile forces generated by the acto-myosin cytoskeleton on cadherin. Therefore, two hypotheses can be proposed to explain the adherens junction displacement during cytokinesis. First, no change of force intensity on cadherin occurs during cytokinesis. This would imply that the force generated by the contractile cytokinetic ring produces plasma membrane ingression by acting exclusively on a structure distinct from the adherens junction. However, this possibility appears unlikely because adherens junctions are required to anchor the cytokinetic ring in Drosophila^18^, although we cannot exclude that this mechanism is different in flies and frogs. Indeed, the interplay of adherens junctions and tight junctions during cytokinesis was recently investigated in Xenopus embryo showing that tight junctions are maintained during cytokinesis^30^. It could be envisaged that cadherin is indeed involved in the stability of cell-cell adhesion to preserve tissue integrity, while another structure undergoes force-drived ingression. Alternatively, imbalanced tensile forces are applied on cadherin during cytokinesis at the site of membrane ingression. It was recently shown that vinculin, an F-actin binding protein which has been used to detect junctional tension *in vivo*^31^, is recruited at the division site^30^. If this imbalanced tensile force on cadherin exists, either force on cadherin is changed unilaterally in the dividing or in the neighboring cell, or force is increased in the dividing cell and reciprocally decreased in the neighboring cells. Taking into account our results, the change would be rather modest in the former case, since it would not exceed the measurement variability (around 1.6 pN). In the latter case, reciprocal force changes would result in an unchanged global tension, as we highlighted. Whether this imbalance is unilateral or bilateral, it will be important to uncover the mechanisms leading to membrane ingression in dividing cells.

Deformation of structure depends not only to instantaneous force intensity but also to its temporal signature. Cadherins are dynamically localized at the adherens junction. Using 3D superresolution quantitative microscopy in Drosophila embryos, it has been shown that E-cadherin clustering is finely regulated through endocytosis and interaction to actin filaments^32^. Using fluorescence recovery after photobleaching measurement in Xenopus embryos, it has been reported that cadherins are stabilized at the cleavage furrow^30^. In that sense, dynamics of cadherin at the plasma membrane could be the primary mechanism leading to membrane deformation.

By using new developments in quantitative fluorescence microscopy to measure spatio-temporal dynamics of molecular tension we present unexpected results where cadherin does not seem to be directly stretched to create membrane deformation during cytokinesis of epithelial cell in vertebrate. Our work paves the way to unveil the molecular mechanisms leading to membrane ingression in vertebrate epithelial dividing cells.

## Materials and Methods

### Plasmids construction and *in vitro* transcription

pT7T-EcadTSMod, pT7T-EcadTL, pT7T-EcadTI, pT7T-CcadTSMod were obtained by PCR amplification of EcadTSMod, EcadTL and EcadTI plasmids (a Kind gift of Dr. N. Borghi) and C-cadherin cDNA (kindly provided by Dr. BM Gumbiner), respectively. PCR products were cloned between BglII and SpeI sites into the pT7T vector using the NEB Gibson assembly kit (NEB). pT7-CcadTSMod was constructed with the NEB Gibson assembly kit following the same scheme as for EcadTSMod: the sequence coding for the 734 first amino acid of C-cadherin were inserted after the BglII site of pT7T, the TS module containing the spider silk protein flanked by mTFP1, EYFP and glycine linker at each side, is inserted just after the first fragment. The last 146 amino acid of C-cadherin were then inserted between TS module and SpeI site of pT7T. Constructs were verified by sequencing. *In-vitro* transcription was performed with mMessage mMachine transcription kit according to manufacturer’s instructions (Ambion) using the EcoRI site for linearization.

### Preparation, microinjection, drug treatment and indirect immunofluorescence of *Xenopus* embryos

*Xenopus laevis* albino adults were obtained from the Biological Resource Centre (CRB, Rennes, France). Embryos were prepared and microinjected as described previously^33^. After microinjection, embryos were kept in ficoll PM400 5% (F4375, Sigma) in F1 (NaCl, 31.25 mM; KCl, 1.75 mM; CaCl_2_, 1.0 mM; MgCl_2_, 0.06 mM; Hepes, 10 mM, pH 7.8^34^), overnight at 14 °C until reaching blastula (stage 9^35^). For imaging experiments, glass slide were prepared with three superposed Gene Frame spacers (AB-0577- Thermoscientific) and filled with ficoll 5%. Embryos were placed in these chambers with vegetal pole facing the glass slide, covered with a glass coverslip and observed at room temperature. For drug treatment, prior observation, embryos were treated with 5 μM Latrunculin A (ab1444290, Abcam) in F1 1x medium for 5 minutes or with 3 mM EGTA (E4378, Sigma) in F1 1x medium without Ca^2+^ for 20 minutes or with 0.5 μM Calyculin A (208851, EMD Millipore) for 90 minutes in F1 1x medium with Ca^2+^. For α-catenin silencing, 8 cells embryo were simultaneously injected with RNA coding for biosensor and with 20 ng of α-catenin morpholino (ATGTTTCCTGTATTGAGAGTCATGC^26^, Genetools) or standard control morpholino (CCTCTTTACCTCAGTTACAATTTATA, Genetools).

For indirect immunofluorescence, embryos were fixed with 2% trichloroacetic acid (TCA) in F1 1X medium for 2 hours at room temperature, devitelinated, permeabilized in PBS plus 1% triton X-100 (PBST 1%) for 10 minutes and saturated one hour in PBST 0.1% plus BSA 1% as previously described^23^. They were then incubated with the following antibodies: anti-GFP (I-16, Santa Cruz, clone 7.1 and 13.1 11 914 460 001, Roche), anti-Xenopus C-cadherin (clone 6B6, DSHB), anti-α-catenin (PA1-25081 Thermo Scientific), anti β-catenin (H102, Sc-7199) or anti-p120 catenin (a kind gift of Dr P McCrea). For actin labelling, embryos were fixed in 4% formaldehyde for 2 hours and treated as described above. Embryos were then incubated with (1.5 U/ml) phalloidin coupled to AlexaFluor 555 (ThermoFisher Scientific) for one hour in PBST 0.1% plus 1% BSA. Embryo were then rinsed and mounted between two spacers (Gene Frame) in Vectashield (Vector).

Fixed imaging was performed using a LSM Leica SP8 confocal microscope equipped with a 63x/1.4 HC PL APO oil immersion objective and laser lines 488 nm, 561 nm. For the detection, hybrid detectors were used. All animal experiments were performed in accordance with the approved protocols and guidelines at Rennes 1 University by the Comité Rennais d’Ethique en Matière d’Expérimentation Animale (C2EA-07) and the French Ministry for Education and Research (3523813).

### Fluorescence lifetime imaging and FRET analysis

All FRET experiments were made using a FastFLIM system (as described previously^24^), using a plan APO 63x oil immersion objective with a 1.4 NA (Leica). For mTFP1 donor excitation, a narrow spectral band was selected (445/23 nm) from a white light laser (Fianium) and sent to the microscope through a dichroic mirror (Di01-T442/514/647, Semrock) inside the spinning head (CSUX1, Yokogawa). The emission of mTFP1 was then filtered using an emission filter on the spinning filter wheel (483/32 nm) and acquired with the intensified timegated CCD camera (Picostar, LaVision and CoolSNAPHQ2, Roper). Five temporal gates of 2.25 ns were acquired sequentially by adjusting step-by-step the delay of the laser signal to trig the gated intensifier. The exposure time of the CCD camera was chosen from 500 ms to 2 s depending on the brightness of the sample. Two to three fields were imaged for each embryo, and for time-lapse acquisition, images were taken every minute. The FLIM calculation was carried out online using “flimager”, a home-made user program developed through MetaMorph (Molecular Devices). All presented fluorescence image correspond to the first temporal gate. Image were then analyzed using ImageJ software (Rasband, W.S., http://rsb.info.nih.gov/ij), thresholded fluorescence images were used to create a mask to eliminate FLIM data out of the membrane, the filtered FLIM image was then used as a mask to retrieve a fluorescence image with only pixel having a non-null FLIM value. Mean FLIM value and mean fluorescence intensity were then obtained from the whole field of the filtered images or along specific lines for kymograph.

For cell division analysis, a region of interest of 7 pixel diameter was used to measure the fluorescence lifetime either at the dividing site, a random position on the proximal membrane of the dividing cell, or a random position on a distal membrane of a non-dividing cell. The ratio of fluorescence lifetime at the various positions was then calculated. The percentage of division progress was assessed by measuring the distance between the two dividing sites at the different time point. Statistical comparison of the FLIM value using the non-parametric willcoxon test, and linear correlation were carried out using R software.

## Additional Information

**How to cite this article:** Herbomel, G. *et al*. Actomyosin-generated tension on cadherin is similar between dividing and non-dividing epithelial cells in early *Xenopus laevis* embryos. *Sci. Rep.* **7**, 45058; doi: 10.1038/srep45058 (2017).

**Publisher's note:** Springer Nature remains neutral with regard to jurisdictional claims in published maps and institutional affiliations.

## Supplementary Material

Supplementary Materials
