# Supplementary material for: Actomyosin-generated tension on cadherin is similar between dividing and non-dividing epithelial cells in early Xenopus laevis embryos
Source: Sci Rep. 2017 Mar 22;7:45058. doi: 10.1038/srep45058 (PMC5361196; doi:10.1038/srep45058)
Supplement: Supplementary Materials [file srep45058-s1.pdf]

Supplemental material

Actomyosin-generated tension on cadherin is similar between dividing and non-dividing epithelial cells in early *Xenopus laevis* embryos.

Gaëtan Herbomel, Guillaume Hatte, Julien Roul, Sergi Padilla-Parra, Jean-Pierre Tassan, Marc Tramier.

Conversion of donor fluorescence lifetime into force intensity

The relation between FRET efficiency and force for the FRET couple Cy3 – Cy5 was recovered from the linear regression of the single molecule measurements presented by Grashoff et al. [13]. as:

(1) $E = -0.072 \times F + 0.5$

In FLIM, the FRET efficiency, E, can be defined, in our case, using the fluorescence lifetime obtained experimentally for mTFP1 alone (τD) and mTFP1 in presence of YFP (τDA), as:

(2) $E = 1 - \frac{\tau_{DA}}{\tau_D}$

In the case where the FRET is maximum, in absence of force, the FRET efficiency for Cy3 – Cy5 couple can be obtained from equation (1) $E_{0,Cy3-Cy5} = 0.5$, for mTFP1-YFP couple, the FRET efficiency might be retrieved from equation (2) with the fluorescence lifetime in presence of acceptor and absence of force corresponding to EcadTL (τDA_0 = 2476 ps) and the fluorescence lifetime of the donor alone obtained with EcadTI (τD = 2724 ps).

In this case, by replacing τD and τDA in equation (2) we have $E_{o,mTFP1-YFP} = 0.091$.

The FRET efficiency depends on the distance between the two fluorescent molecules, which is specific for each FRET couple and can be expressed as:

(3) $E = \frac{1}{1+\left(\frac{R1}{R0}\right)^6}$

In which R0 is a constant that depends on the fluorescent protein couple and R1 is a variable related to the distance between the two fluorescent proteins. Using this equation, R1 can be expressed as:

(4) $R1 = R0 \times \sqrt[6]{\left(\frac{1}{E} - 1\right)}$

Using this equation, in absence of force, for $E_{0,Cy3-Cy5} = 0.5$ we have $R1_0$ = R0, and for $E_{0,mTFP1-YFP} = 0.091$, we have $R1_0 = 1.467 \times R0$.

R0 for mTFP1 – YFP FRET couple has been defined to be $R0 = 6\ nm$ by Grashoff, et al. 2010.

So, in our case, $R1_0 = 8.804\,nm$, which correspond to $R1_0 = R0 + 2.804$. Then we are able to correct the changes of fluorescent protein couple in the equations, in presence of force when the FRET efficiency, E, varies.

$$(5)\quad R1 = R0 \times \sqrt[6]{\left(\frac{1}{E} - 1\right)} + 2.804$$

By combining the FRET efficiency expression (2), R1 expression (5) and the equation of FRET efficiency versus force (1) into equation (3) we have:

$$(6)\quad 1 - \frac{\tau DA}{\tau D} = \frac{1}{1 + \left[\frac{R0 \times \sqrt[6]{\left(\frac{1}{(-0.072\,F + 0.5)} - 1\right)} + 2.804}{R0}\right]^6}$$

The force, corrected for the fluorescent protein couple and expressed for fluorescence lifetime might then be expressed as:

$$(7)\quad F = \left(-\frac{1}{0.072}\right) \times \left[\frac{1}{\left(\sqrt[6]{\left(\frac{1}{1 - \frac{\tau DA}{\tau D}} - 1\right)} - \frac{2.804}{R0}\right)^6 + 1} - 0.5\right] (5)$$

Knowing τD, which is the fluorescence lifetime of EcadTI (2724 ps), and R0 = 6 nm, we are able to trace a calibration curve using a wide range of fluorescence lifetime for τDA. The calibration curve for EcadTSMod is presented in Fig. S3a. The equation of this curve allows us to directly convert into force the corresponding fluorescence lifetime obtained experimentally.

Supplemental Figures

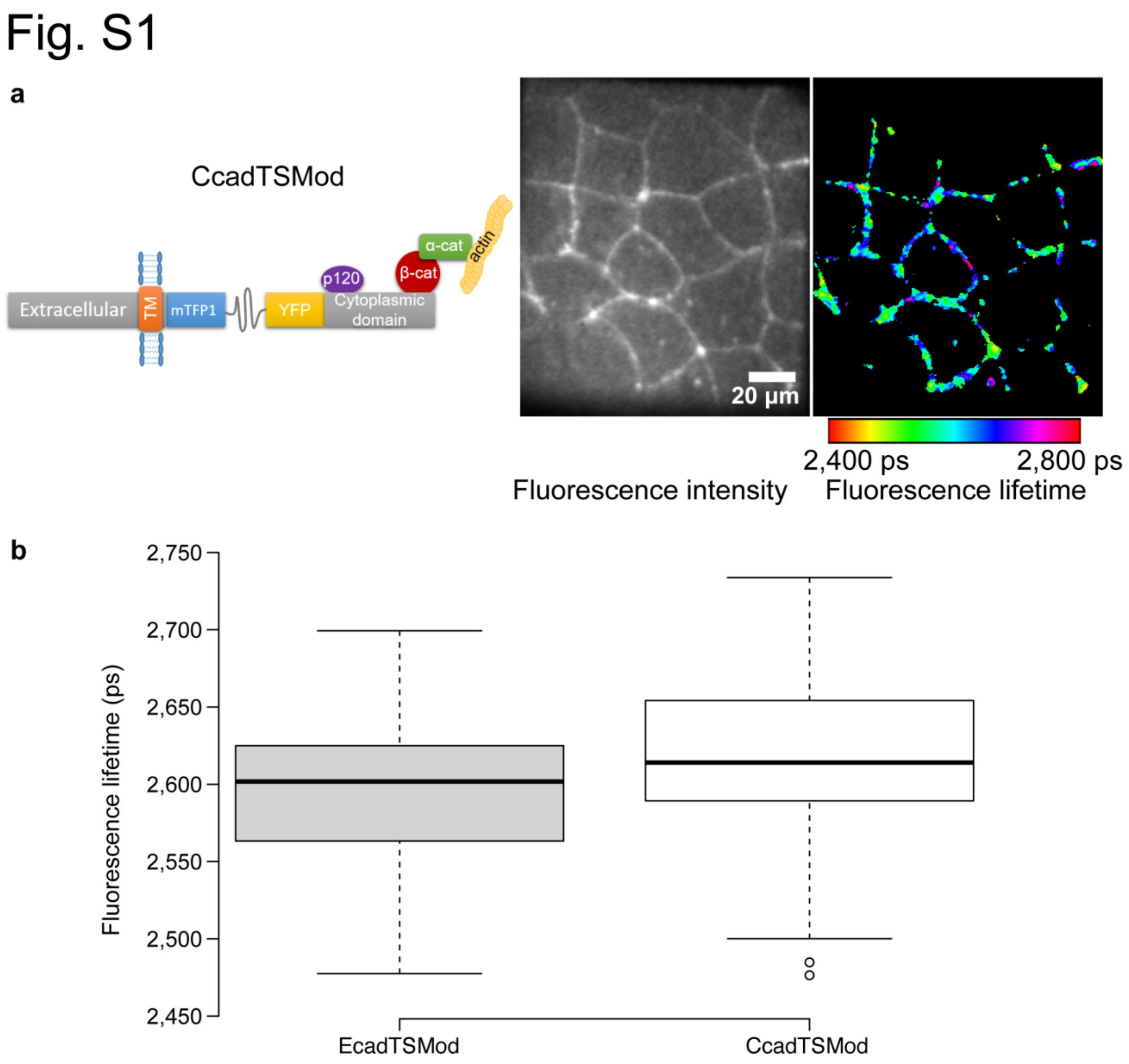

<u>Fig. S1</u>: Localization and fluorescence lifetime of CcadTSMod biosensor. **a**. Schematic representation of CcadTSMod construct (left) and its localization in living Xenopus blastula (middle) with the corresponding fluorescence lifetime image (right). **b**. Boxplot of EcadTSMod and CcadTSMod mean fluorescence lifetimes calcuted with respectively 105 and 134 independent acquisitions (representing for CcadTSMod: 15 independent experiments and 41 embryos).

Fig. S2

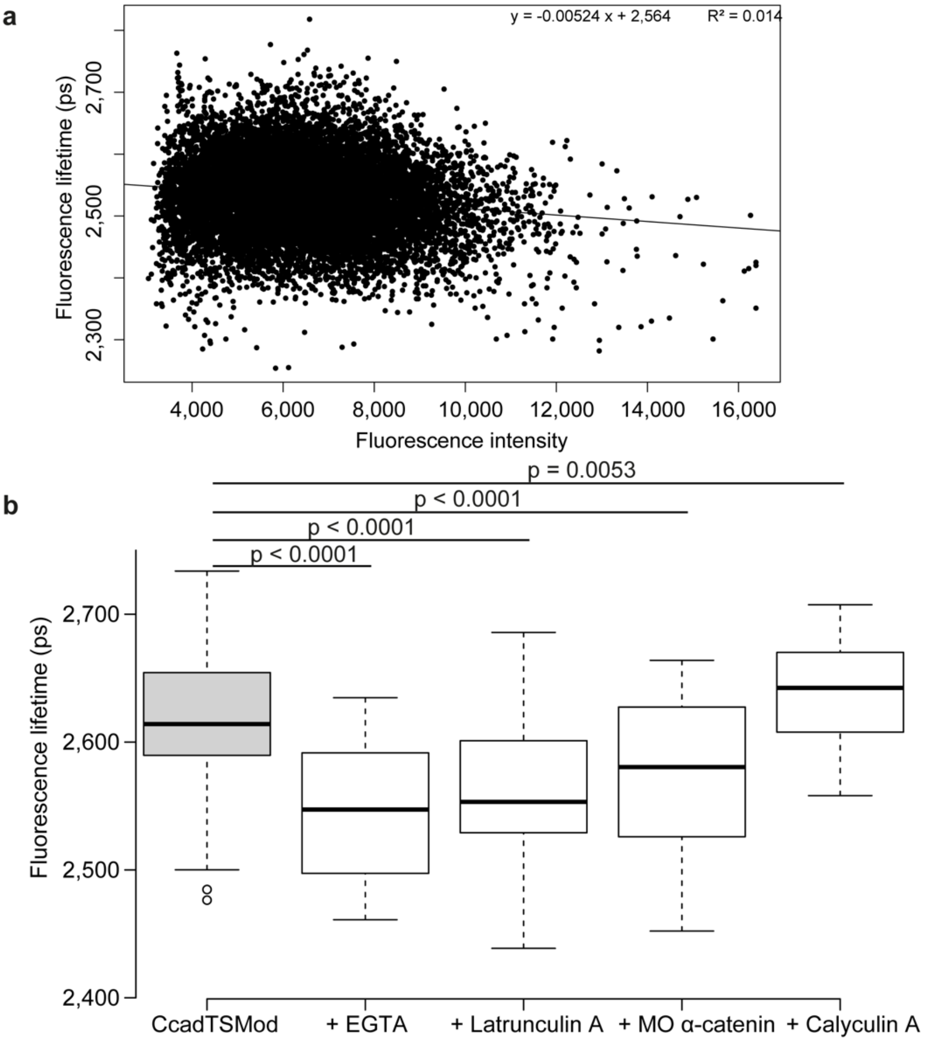

Fig. S2: CcadTSMod is integrated and responsive to various stimuli. **a**. Representative scatter plot of the fluorescence lifetime vs fluorescence intensity for each pixel of an acquisition. Equation of the linear regression is specified in the top right corner. The average equation for the 14 independent experiment is $y = -0.0053\,x + 2630$ with a R^2 = 0.0163. **b**. Boxplot of the mean fluorescence lifetime of CcadTSMod in untreated embryos and after EGTA, latrunculin A, morpholino against α-catenin or Calyculin A treatment, with respective number of acquisitions N= 134, 27, 45, 65 and 41 (respective number of experiments: 15, 3, 4, 4 and 2; respective number of embryos: 41, 9, 16, 21 and 20). For each comparison, the p value is indicated. Dots correspond to outliers.

Fig. S3

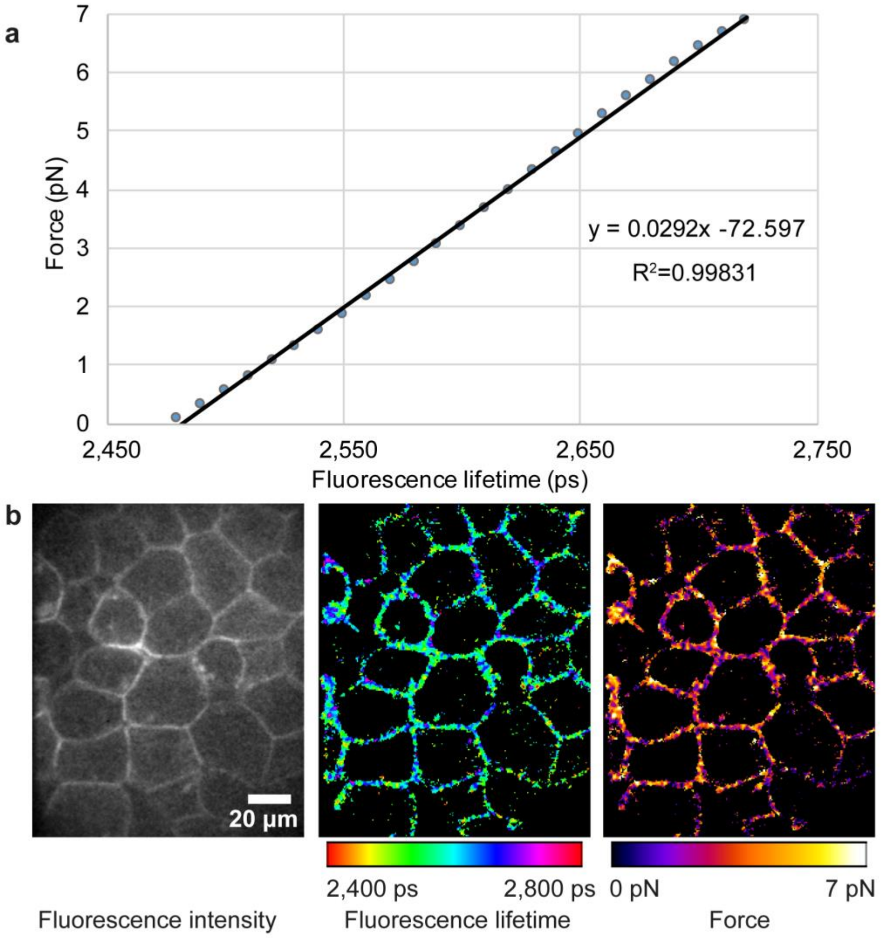

<u>Fig. S3</u>: The fluorescence lifetime can be directly converted into a force value. **a**. graphical representation of the linear relation between fluorescence lifetime and force, obtained as described in supplementary material. Equation of the linear regression presented was used to convert fluorescence lifetime into force. **b**. EcadTSMod fluorescence image in living Xenopus embryo (left) and the corresponding fluorescence lifetime image (center) and force image (right).

Fig. S4

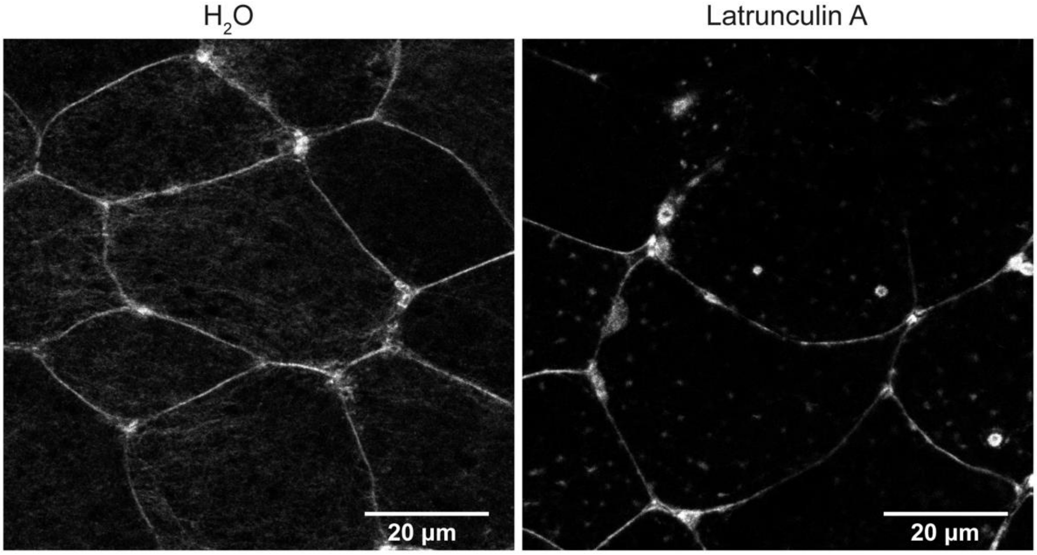

<u>Fig. S4</u>: Actin cytoskeleton is disrupted by latrunculin A treatment. Xenopus laevis embryos were treated with 5 µM latrunculin A during 20 minutes (latrunculin A) or not (H_2O). Embryo were fixed and actin filaments were labelled using Alexa Fluor 568 phalloidin.

Fig. S5

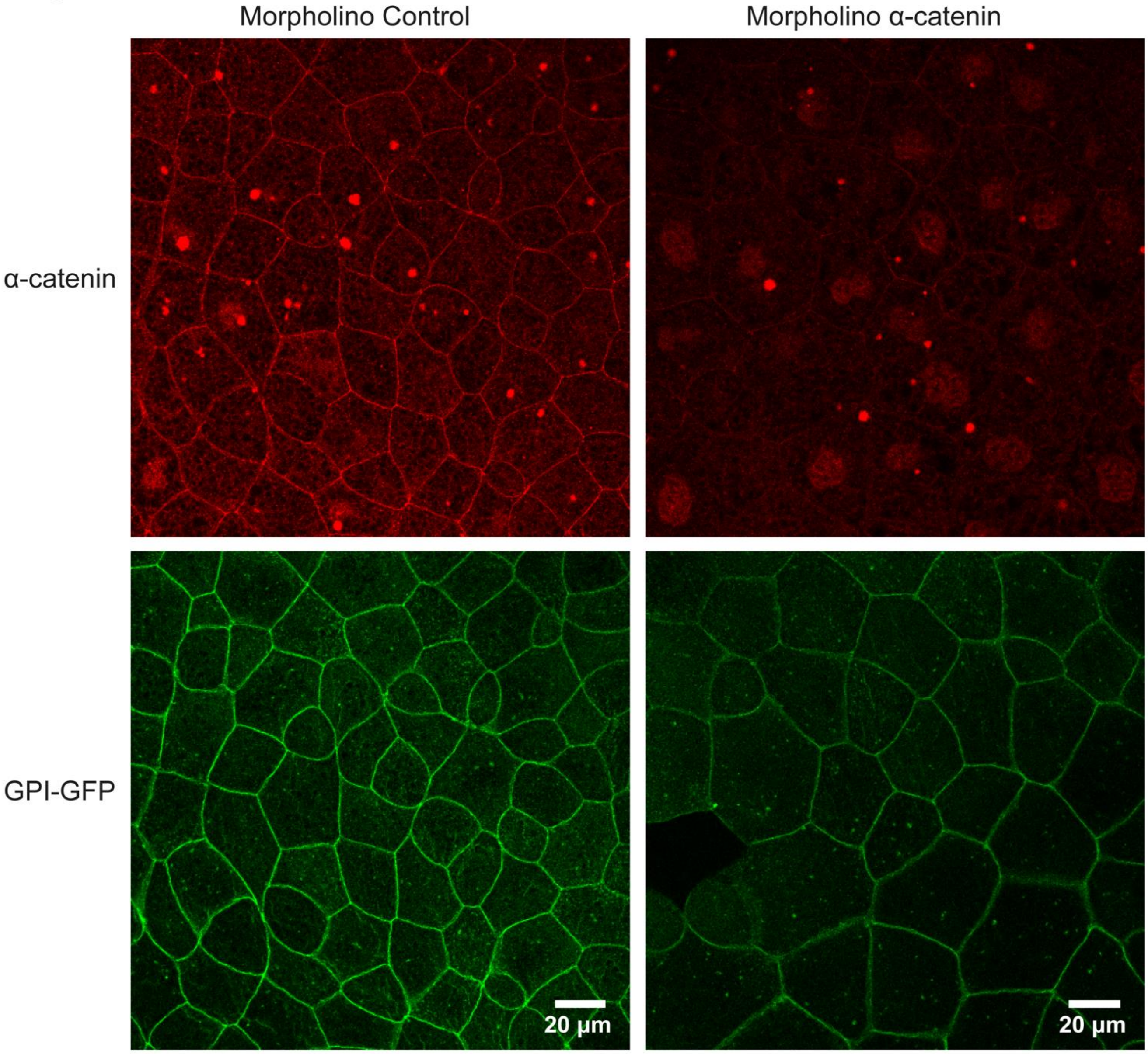

<u>Fig. S5</u>: α-catenin is depleted after injection of morpholino against α-catenin. Embryos were microinjected with either a Morpholino against α-catenin or a control Morpholino together with GFP-GPI mRNA. GFP-GPI which labels plasma membrane, is used as a tracer. When embryos reached developmental stage 10, they were fixed in TCA 2% and processed for indirect immunofluorescence to detect α-catenin (red) and GFP-GPI (green) was detected by indirect immunofluorescence using anti-GFP antibody.

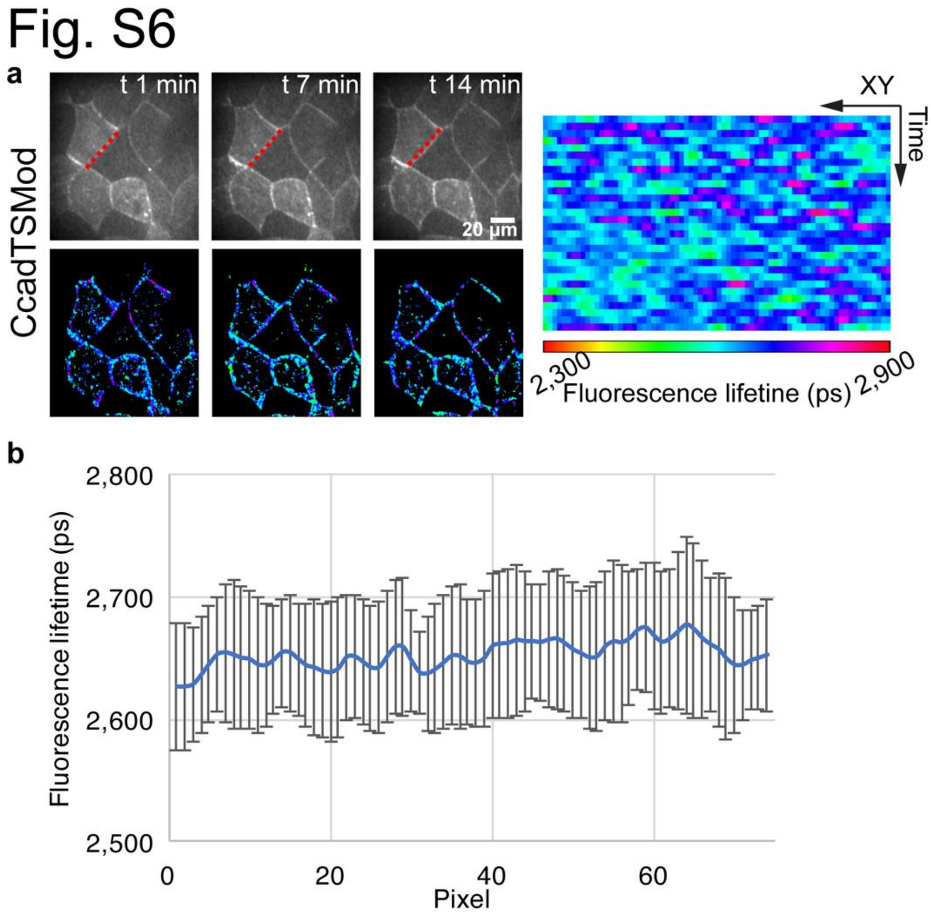

Fig. S6: Spatio-temporal analysis of CcadTSMod biosensor in the Xenopus embryo. **a**. Temporal acquisition of CcadTSMod in living Xenopus embryo at blastula. Fluorescence images are presented on the upper row, the lower row correspond to the measured fluorescence lifetime images. A kymograph of the whole temporal acquisition is represented in the right part, the corresponding XY coordinates are shown by the red dotted line in fluorescence images. **b**. CcadTSMod mean fluorescence lifetimes and standard deviations of al XY coordinates over the time series. Values represent the mean ± SD.

Fig. S7

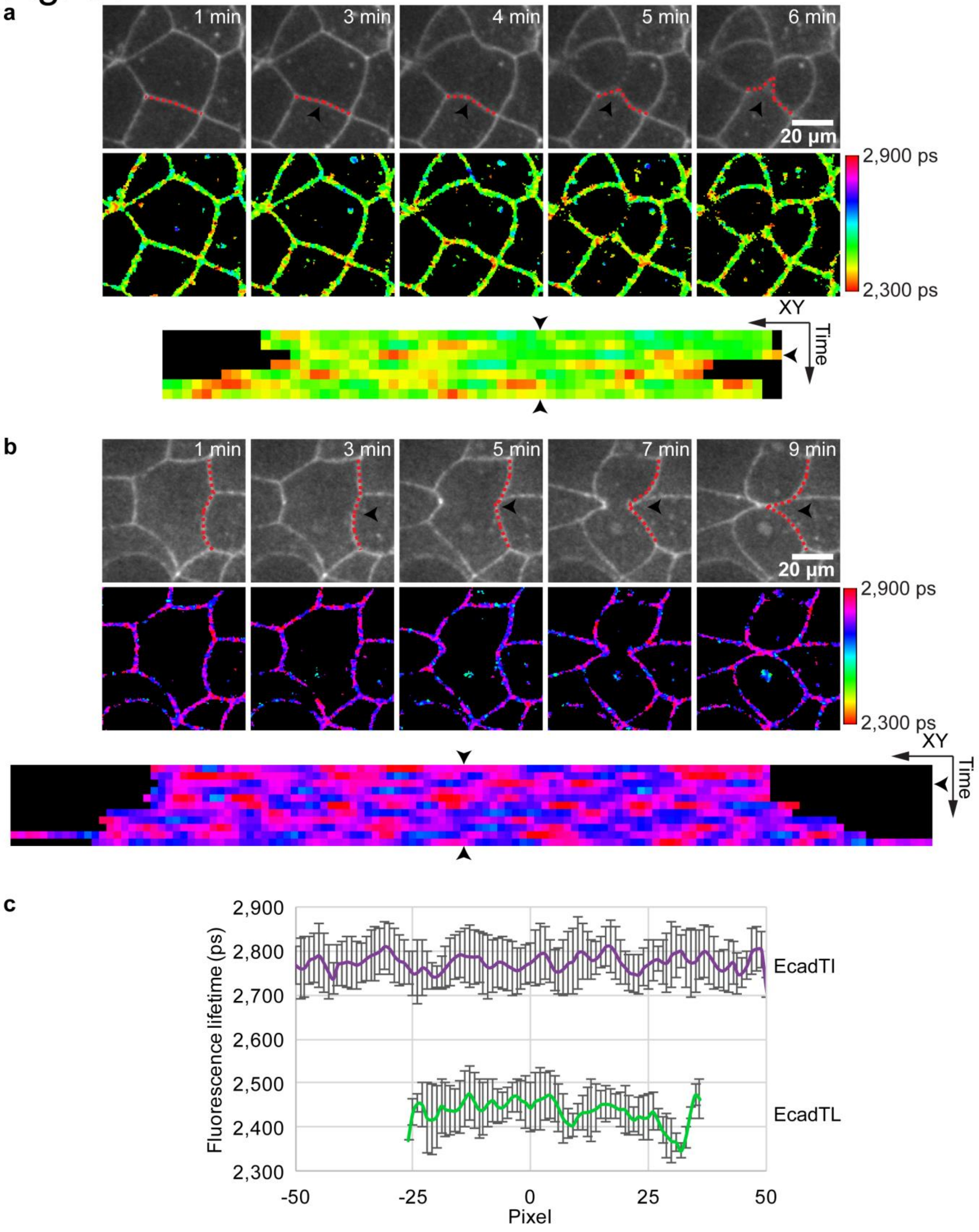

<u>Fig. S7</u>: Fluorescence lifetime of EcadTL and EcadTI is stable during cell division. Representative spatio-temporal acquisition of EcadTL (**a**) and EcadTI (**b**) fluorescence images and the corresponding fluorescence lifetime images at different time points. On fluorescence images, the black arrowheads point to the division site and the red dotted lines indicate the cell-cell contact presented as a kymograph below. The temporal resolution is 1 image every minute. The vertical arrowheads on the

kymograph indicate the site of division while the horizontal one marks the starting time of ingression. The length of the kymograph varies over time due to the stretching of the membrane during the division. The images are representative of 7 and 8 junctions respectively for EcadTL and EcadTI. **c**. Mean fluorescence lifetime and standard deviations of all XY coordinates over the time series for EcadTL (green) and EcadTI (purple) in dividing Xenopus embryo epithelial cells. The pixel axis is centered on the division site. Values represent the mean ± SD.

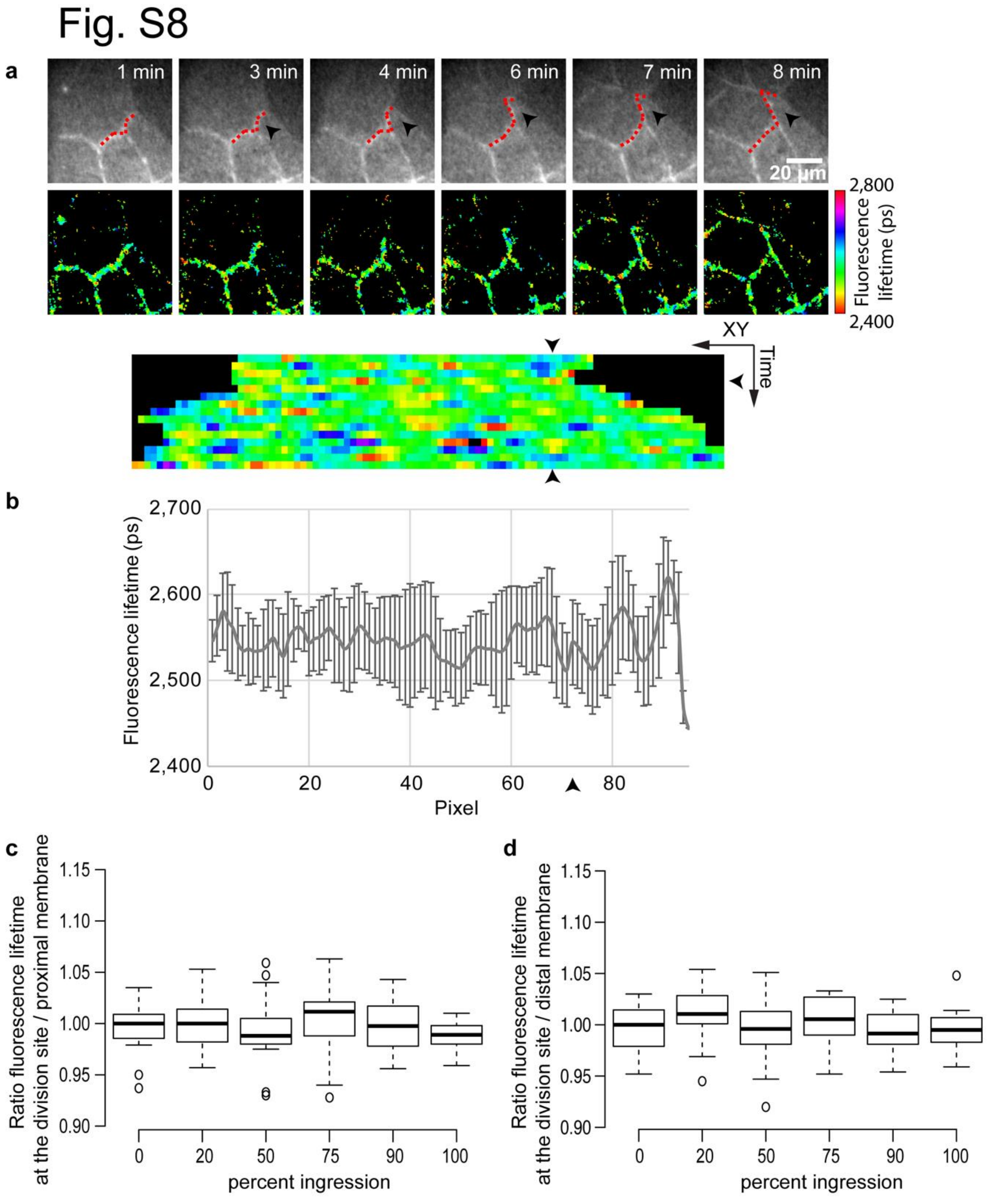

Fig. S8: Forces applied to CcadTSMod are stable during epithelial cell division. a. Spatio-temporal acquisition of CcadTSMod fluorescence images and the corresponding fluorescence lifetime images at different time points during cell division. On fluorescence images, the black arrowhead points to the division site and the red dotted line indicates the cell-cell contact presented as a kymograph below. The temporal resolution is 1 image every minute. The vertical arrowheads on the kymograph indicate the site of division while the horizontal one marks the starting time of ingression. The length of the kymograph varies over time due to the stretching of the membrane during the division. **b**.

Mean fluorescence lifetime and standard deviations of all XY coordinates over the time series for CcadTSMod in dividing Xenopus embryo epithelial cells. Values represent the mean ± SD. **c** and **d.** Ratio of the fluorescence lifetime measured at the dividing site and on the proximal membrane (**c**) or on a distal membrane on a non-dividing cell (**d**), during the progress of cell division. The fluorescence lifetime is measured at the same time point at the dividing site and in a region in the proximal or distal membrane, the corresponding percentage of progress of division is calculated by measuring the distance of the two dividing point. The graph shows the results obtained for 16 division sites (which represents 7 independent experiments, 10 embryos and 11 cells). Dots corresponds to outliers.
